# Localization and Tracking of Implantable Biomedical Sensors

**DOI:** 10.3390/s17030583

**Published:** 2017-03-13

**Authors:** Ilknur Umay, Barış Fidan, Billur Barshan

**Affiliations:** 1 Department of Mechanical and Mechatronics Engineering, University of Waterloo, 200 University Ave W, Waterloo, ON N2L 3G1, Canada; iumay@uwaterloo.ca; 2 Department of Electrical and Electronics Engineering, Bilkent University, Bilkent, Ankara TR-06800, Turkey; billur@ee.bilkent.edu.tr

**Keywords:** localization, tracking, biomedical robotics, gastrointestinal (GI) tract, capsule endoscopy, biomedical microrobot, wireless capsule endoscopy

## Abstract

Implantable sensor systems are effective tools for biomedical diagnosis, visualization and treatment of various health conditions, attracting the interest of researchers, as well as healthcare practitioners. These systems efficiently and conveniently provide essential data of the body part being diagnosed, such as gastrointestinal (temperature, pH, pressure) parameter values, blood glucose and pressure levels and electrocardiogram data. Such data are first transmitted from the implantable sensor units to an external receiver node or network and then to a central monitoring and control (computer) unit for analysis, diagnosis and/or treatment. Implantable sensor units are typically in the form of mobile microrobotic capsules or implanted stationary (body-fixed) units. In particular, capsule-based systems have attracted significant research interest recently, with a variety of applications, including endoscopy, microsurgery, drug delivery and biopsy. In such implantable sensor systems, one of the most challenging problems is the accurate localization and tracking of the microrobotic sensor unit (e.g., robotic capsule) inside the human body. This article presents a literature review of the existing localization and tracking techniques for robotic implantable sensor systems with their merits and limitations and possible solutions of the proposed localization methods. The article also provides a brief discussion on the connection and cooperation of such techniques with wearable biomedical sensor systems.

## 1. Introduction

Recent discoveries in electronics, nanotechnology, semiconductor technology and advances in material science have resulted in promising new approaches for the development of medical devices. As a result, medical innovation leading to lower cost of healthcare, minimally-invasive procedures and shorter recovery times has become equally or comparably important to healthcare business leaders, educators, clinicians and policy makers. Miniaturization of large electronic components has especially enabled the production of sufficiently small implantable or wearable biomedical sensor systems, such as smart pills or capsules, pacemakers and body sensor networks. Those smaller and low-cost wireless biomedical devices are more convenient for implanting inside the human body or for wearing.

**Figure 1:**
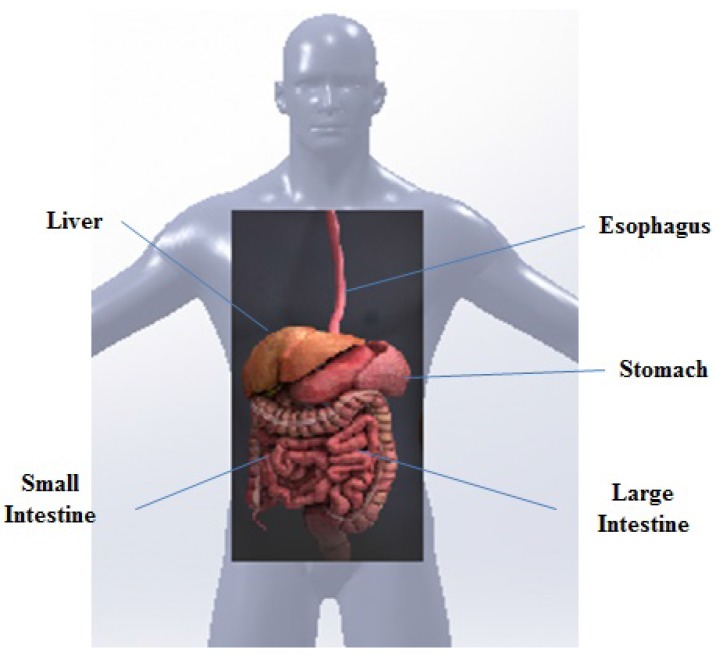
GI tract of the human body.

Wireless capsule endoscopy (WCE) systems constitute an important class of the aforementioned biomedical systems [1]. The interest in WCE has increased with the effect of noticeable gastrointestinal (GI) disease statistics. Every year, in the USA, about 150,000 additional patients suffer from colorectal cancer, and about 60,000 die from the disease. More than 230,000 (one in every 150) Canadians suffer from inflammatory bowel disease (IBD) every year. Today, diagnosis and treatment of GI tract (Figure 1) health problems, such as Crohn’s disease, chronic diarrhoea, obscure bleeding, irritable bowel syndrome and colon cancer, are extremely challenging problems for physicians [2,3]. Conventional diagnosis and treatment methods, such as endoscopy and colonoscopy, are often painful and uncomfortable for patients because of the difficulty of accessing various parts of the GI tract [4]. WCE overcomes this problem, replacing the inconvenient endoscopic tools with swallowable wireless endoscopic capsules (WECs).

**Figure 2:**
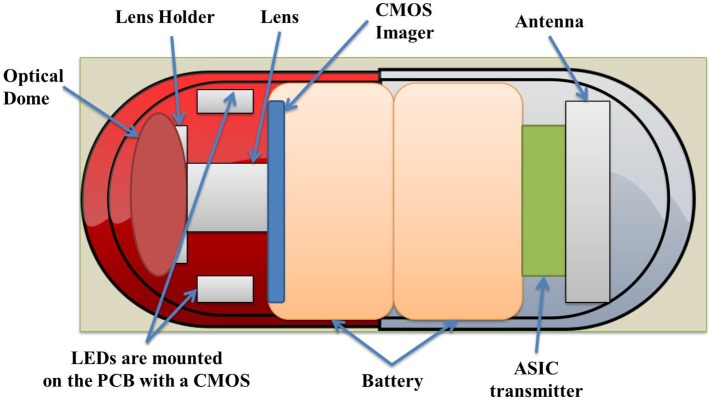
Structure of a typical wireless endoscopic capsule (WCE), based on the architecture of the M2A capsule.

**Figure 3:**
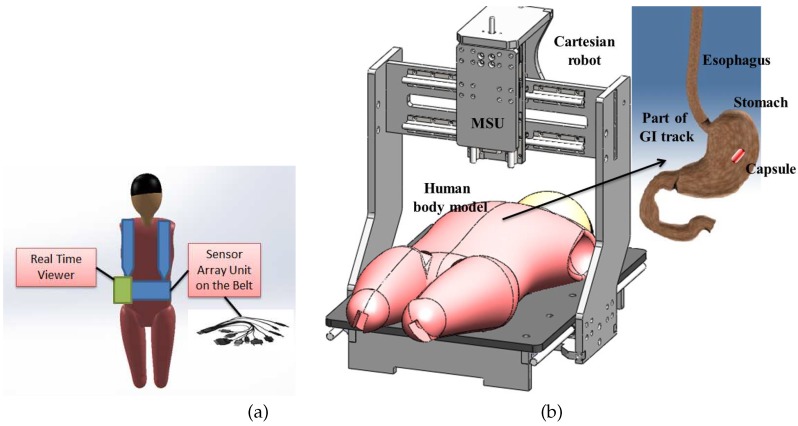
(**a**) WCE receiver set; (**b**) a robotic navigation system for gastric capsule endoscopy.

WCE starts with the patient swallowing the WEC. The natural peristalsis force of the human body helps the WEC to move through the GI tract without any harm or pain, collecting images and other data and transmitting them to a monitoring system placed outside the body. GI physiological parameters, such as temperature, pressure or pH level, can be measured by WECs. Figure 2 illustrates a typical WEC, based on the architecture of the M2A capsule [5], which is composed of an image sensor, a radio frequency (RF) data transmitter, an illumination unit and a battery. A typical WCE system comprises a spatial robot manipulator with a sensor unit attached to its end effector or a belt-shaped sensor array unit attached to the body with a real-time viewer. A sensor array unit concept attached to the body with a real-time viewer [4] and a 3D Cartesian robot concept [6] for manipulating the sensor unit are illustrated in Figure 3a,b, respectively. Other examples of spatial robots for manipulating such sensor units are reported in [7].

The WEC swallowing approach was first proposed in the preliminary communication article [8] on RF transmission of temperature and pressure data from the human GI tract. Currently, WCE has been established as a safe and convenient tool for the diagnosis and treatment of GI tract diseases and disorders, including GI bleeding, small intestine tumours and Crohn’s and Celiac diseases. It is indicated that over 1,250,000 patients have benefited from the WCE test all around the world [7,9]. These statistics demonstrate the acceptance and importance of WCE technologies in the diagnosis and monitoring of GI diseases. A detailed review of WCE technologies is provided in [4,7].

PillCam (SB3, Colon2, UGI, PATENCY) WCEs constitute the pioneer group of commercially available WCE products produced by Given Imaging Ltd. and currently marketed by Medtronic Inc. SmartPill by Given Imaging Ltd., EndoCapsule by Olympus Co., MiroCam by IntroMedic Co., OMOM capsule by Chongqing Jinshan Science and Technology Co. and the CapsoCam panoramic HD imaging capsule by CapsoVision Inc. are the other key commercial WCE products produced so far.

In the commercial localization technologies used in the M2A WEC by Given Imaging Ltd., a set of receivers is positioned on the patient’s abdomen, and a transmitter inside the WEC sends data to the receivers. Location data are calculated based on the principle that the receiver closest to the capsule receives the strongest signal [7,10]. This approach is not used widely because of its low accuracy (3.77 cm) [7]. The motility monitoring system (MTS2) by Motilis Medica SA provides data for visualizing regional transit time and WEC location. SmartPill by Given Imaging Ltd. provides pressure, pH level and temperature data to evaluate the GI track conditions. The aforementioned localization technologies in use are attractive since they are simple and do not require additional equipment [11]. However, they suffer from exterior electromagnetic noise and complicated RF signal absorption characteristics of the human body [12]. Further, these technologies still do not provide accurate location and orientation data of the capsule associated with problems, such as tumour diagnosis [3,4].

Acquiring accurate data on the capsule’s location and orientation while the capsule moves along the GI track is one of the most crucial problems for several reasons: (1) capsule position does also provide information on the location of tumours, bleeding or other problematic issues in the GI tract; (2) without position information, finding solutions to other problems of capsule endoscopy (CE), e.g., tracking of the capsule or arranging the working time of the capsule for potential targeted drug delivery, and adapting the frame rate for video transmission, is nearly impossible; (3) it is helpful to determine the insertion path of the biomedical device to eliminate the repetitive attempts of invasive endoscopy; (4) localization is essential in developing effective actuation systems; (5) location- and orientation-based path reconstruction enables various micro-robotic surgeries and reveals the uncertain interior small intestine environment to researchers for educational objectives; (6) precise localization enables transmission power control and energy saving by turning the device on and off. Because of the complex and non-homogeneous medium of the interior of the body, 3D restoration of the WCE acting route in the small intestine is still in its start-up phase [4,7,13,14]. Currently, most commercial software packages only provide 2D tracking of the capsule route [14]. Therefore, there is a need for further research in localization technologies and algorithms of WCE.

As an example of implantable medical sensor applications outside the WCE field, implantable bladder sensors are applied to patients who suffer from losing urinary bladder control/sensation, also known as urinary incontinence (UI). They provide direct measurement of the bladder urine volume or pressure for long-term monitoring by eliminating the risks of infection caused by catheters, wires or high-energy waves. In implantable bladder sensors, hermiticity, bio-compatibility, drifting, telemetry, power transfer and compatibility issues still require more research for enhanced patient comfort and long-term monitoring. The study [15] indicates that the wireless communication distance is an essential factor for such sensors, since RF signals rapidly spread in the human body, but inductive coupling necessitates alignment and appropriate localization of both interior and exterior coils for effective power transmission.

As a second example, robotic transapical transcatheter aortic valve implementation (TA-TAVI) devices are extremely helpful for the diagnosis and treatment of heart diseases [16,17]. In recent advancements, one of the challenging issues for TAVI is the localization and tracking problem of the valve during the cardiovascular procedure. Researchers focus on improving the 2D valve localization of TAVI, integrating a robotically-activated delivery sheet and ultrasound or computed tomography or magnetic resonance imaging (MRI) and ultrasound techniques for TA-TAVI. However, there are numerous limitations and challenges of each of these approaches that require further investigation.

In the research domain, four main approaches have been explored for biomedical implantable sensor localization: (1) electromagnetic wave-based techniques; (2) magnetic field strength (MFS)-based techniques; (3) hybrid techniques; and (4) others, as shown in Table 1.

The purpose of this article is to provide a literature review on the techniques and technologies to localize and track biomedical sensors inside the human body. The rest of the article is organized as follows: In Section 2, we summarize the existing RF electromagnetic signal-based localization techniques and algorithms, as well as the challenges in CE in the literature. The details of the magnetic signal-based techniques are provided in Section 3. Section 4 provides a literature review of the distance and/or bearing measurement-based location estimation algorithms utilized in the localization schemes in Section 2 and Section 3. Section 5 and Section 6 introduce the hybrid and other techniques used in biomedical sensor localization, respectively. Section 7 presents a discussion on extensions and counterparts of the covered localization techniques for wearable biomedical sensor systems. A summary and concluding remarks are provided in Section 8.

## 2. Radio-Frequency Electromagnetic Signal-Based Localization and Tracking

The main advantages of electromagnetic wave-based approaches are that (i) the electromagnetic signal radiated by the wireless biomedical sensor (WBS) can be used without any need for additional equipment or signal generation and (ii) these approaches are not affected by the magnetic field used for actuating the WBS, unlike magnetic strength-based localization techniques [6,7,47]. On the downside, high-frequency electromagnetic waves have much higher attenuation as compared to magnetic waves when they propagate through human tissue, and low-frequency electromagnetic waves provide a low precision of localization [7,19]. In the literature, there are various electromagnetic wave-based localization technologies for localization and tracking of a WBS inside the human body, including received signal strength (RSS), time of flight (ToF), time difference of arrival (TDoA), angle of arrival (AoA) and RF identification (RFID)-based methodologies [7,22].

It is indicated in [7] that for the aforementioned near-field applications, the time-based ToF and TDoA techniques are unrealizable due to the high speed of radio waves ($3\times 10^{8}$ m/s); therefore, highly (nanosecond level) accurate synchronized clocks are required to provide a localization resolution of 30 cm. Similarly, AoA techniques are inappropriate in the GI tract conditions because of their low level of accuracy in indoor environments [7]. Although many positioning techniques have been introduced, none of the mentioned studies could provide an absolute solution to resolve the WBS positioning issue. Between these techniques, RF signal-based positioning methods have certain merits of application and require a lower cost of implementation. Thus, those methods have already been preferred in several commercial wireless biomedical capsules (WBC), such as SmartPill, MicroCam and the M2A [4,7].

Unique problems exist for localization inside the human body, because of its complicated structure: the shadowing effects, variable and uncertain signal propagation velocities and path loss parameters in the whole human body, strong absorption of human tissue and the peristalsis movement. Furthermore, detailed RSS and ToF models are fairly complex, since the signals received from the body-mounted sensors are distorted due to multi-path effects caused by the refraction at the boundary of human organs and tissues [7,49,55,56].

In addition to the technological challenges mentioned above, another essential point to take into account in localization system design for WBSs is the regulatory safety standards [4,57,58]. The band or power level of the signals to be used for biomedical sensor localization are upper-bounded by such standards, e.g., the Medical Implant Communication Service (MICS) standard asserts use of the 402–405 MHz frequency band for communication with medical implants [4,58]. In order to decrease the interference among signals within the allowed band, the channel bandwidth is limited to 300 kHz. Therefore, having a high data rate is not easy [4,7,57,59]. The aforementioned band limitations further lead to limitations in MICS signal transmission power and accuracy degradation in ToF measurements. Another aspect of safety limitations is the power absorption characteristics of human tissues exposed to electromagnetic signals emitted by WECs. A detailed analysis of this aspect is provided in [60].

The standard perspective for RF-based positioning utilizes a two-step estimation procedure to find the position. The first step is to guess the environmental coefficients that are related to the transmitter position, such as relative permittivity for ToF or path loss coefficient for RSS-based techniques, with a priori data on the environmental coefficient of each organ or medium. The second step uses these estimated parameters to subsequently guess the position based on an appropriate localization and tracking algorithm [4,6,7,22,26,47,61,62].

### 2.1. RSS Based Techniques

RSS or RSS indicator (RSSI) is a distance measurement method that depends on the signal strength sensed by a receiver placed in the sensor [63,64]. In a general RSS model, the target signal source *T*, which needs to be localized, emits a pulse with original power $P_{T}$. The power $P_{S}$ received by the receiver *S* has an exponential decay model, which is a function of $P_{T}$, the distance $d_{T}$ between *S* and *T*, and the path loss coefficient (exponent) (*η*) represents the signal propagation effect in the corresponding environment. The widely-accepted mathematical model is:

(1) $$P_{S}=K_{\ell }P_{T}d_{T}^{-\eta },$$

where $K_{\ell }$ represents the other factors, such as the influences of antenna height and gain. $K_{\ell }$ is taken into consideration as log-normal, and in most cases, it is ignored in the algorithm resulting in lower cost and the simplified model:

(2) $$P_{S}=P_{T}d_{T}^{-\eta }.$$

The RSS techniques generally provide lower cost among all existing radio technologies, such as Wi-Fi and ZigBee. However, RSS can suffer from multi-path influences, such as shadowing, reflection, diffraction and refraction due to unpredictable environmental conditions, especially for indoor applications [64]. In modelling, these influences are also lumped and included in the coefficient $K_{\ell }$ of Equation (1).

**Figure 4:**
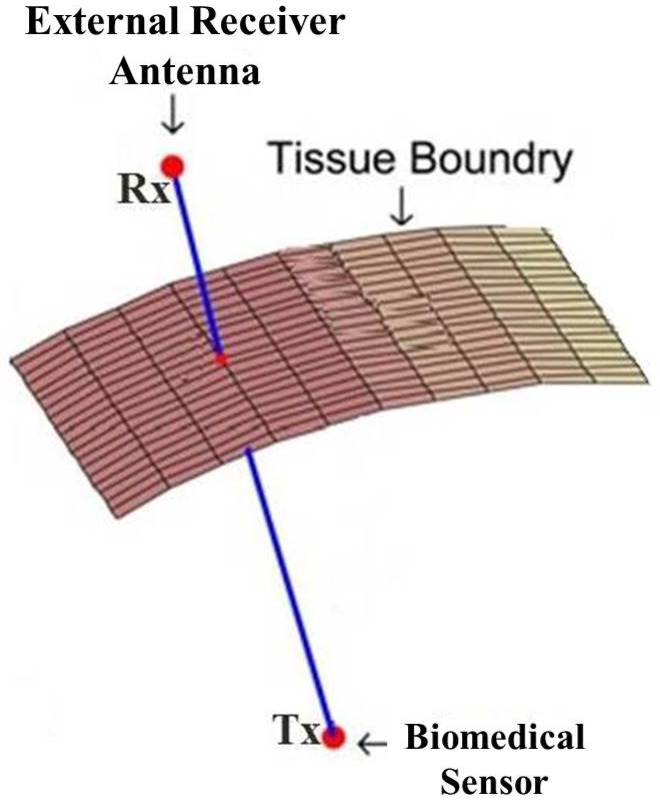
Signal transmission through tissue layers inside the human body; Tx: transmitter and Rx: receiver.

The study in [12] is one of the first on developing an RSS-based WEC localization system. The localization system developed in [12] is based on measuring the RSS of a WEC’s wireless transmission data via eight exterior antennas, and it has been utilized in Given Imaging Ltd.’s M2A capsule. RSS-based methods fuse the power measurement of the signals received at different positions on the abdomen for the localization of the WEC (Figure 4). Usually, a signal propagation model is used, which relates the RSS to the distance between the in-body transmitter and the receiver located on the body [4,7,12,18,22,61]. In RSS-based localization systems, the use of transmitter-receiver pairs allows transmitting a signal from the biomedical sensor to some receivers placed on the abdomen and having those receivers provide the signal strength measurements to be used in determining the correct location of the object [18].

After the distances from the receiver are estimated, a trilateration method could be employed to calculate the coordinates of the WEC. In [21], instead of using a propagation model, the authors used an algorithm based on a look-up table, which stores the off-line measurements carried for different WEC positions. Later on, during the experiment, the measured RSS was compared with the look-up table entries to find the closest value, and the corresponding WEC position in the table was taken as the position estimate.

There have been efforts to build a more accurate propagation model, which depends on not only the distance, but also the antenna orientation and tissue absorption [19,20]. Thus, the RSS-based methods need a propagation model that varies from person to person due to the complex radio wave absorption properties of the human tissue [7]. The authors in [23] have introduced an algorithm based on a look-up table, storing the previous 2D positions of a biomedical sensor to be localized, together with the corresponding signal strength values. During the experiments of [23], the parameters in the look-up table were checked against the newly-acquired dataset to determine the nearest equivalent and select the most likely location.

The studies in [19,20] consider both the distance based on the RSS data and the effects of the antenna orientation factors and tissue absorption impacts to develop an attenuation compensation model. The researchers in [24,25] take into account the effect of variant organs and sensor array’s geometry on the location error in positioning systems based on the signal strength.

### 2.2. ToF-Based Techniques

**Figure 5:**
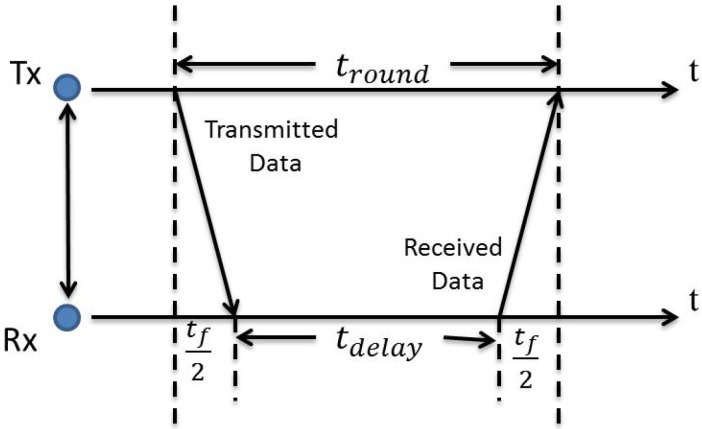
Principle of the two-way ToF distance measurement.

In ToF-based techniques, the distance measurement sensor unit consists of a transmitter, receiver and precision clock; the transmitter transmits a signal, which is reflected by a biomedical sensor and received by the receiver, and the ToF reading is used to estimate the distance. The environmental conditions are shown in the electromagnetic signal propagation velocity

(3) $$v=\frac{c}{\sqrt{\varepsilon }},$$

where *c* is the speed of light and *ε* is the propagation coefficient. The distance $d_{T}$ is estimated by multiplying this propagation velocity and the sensed ToF value. The ersatz mathematical model is illustrated in Figure 5 and can be formulated [65] as

(4) $$t_{f}=\frac{2d_{T}}{v_{ave}}=d_{T}\sqrt{\bar{\varepsilon }}$$

where:

$$\bar{\varepsilon }=\frac{4\varepsilon }{c^{2}}=\frac{4}{v_{ave}^{2}},$$

The $v_{ave}$ is the average signal propagation velocity; $t_{f}$ represents the single-trip propagation time between the target node and the reference node; $t_{delay}$ denotes to the processing time at the reference node; and $t_{round}=t_{delay}+t_{f}$ denotes the round-trip propagation time of impulses.

A largely accepted merit of ToF-based methods is their high precision compared to RSS-based methods [4,7,22]. ToF-based methods consider the signals’ travelling times between the known sensor nodes and unknown target nodes. Ranging data are estimated by multiplying the propagation velocity of the RF signal and the measured ToF value. The ToF value can be detected not only by sensing the phase of the received narrow-band carrier signal, but also by directly detecting the arrival time of a wide-band narrow signal [4,18]. However, the study in [49] shows that time-based methods need strict time synchronization and a high bandwidth to achieve the desired precision, which is difficult to achieve in the MedRadio band (401–406 MHz). It could be used for ultra-wide band (UWB)-based localization [66].

**Figure 6:**
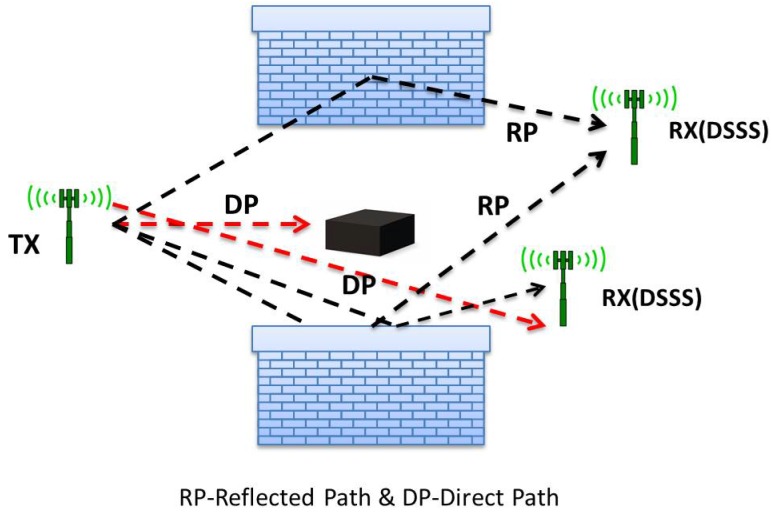
DLoS and DSSS scenarios for ToF ranging.

There exist three widely-known techniques for ToF-based localization. Firstly, direct line of sight (DLoS) can provide higher accuracy for outdoor applications. However, considerably large measurement errors can be observed due to the severe multipath environment for indoor applications. It is a direct function of the distance between the transmitter and the receiver. Secondly, the direct sequence spread spectrum (DSSS) demonstrates better performance for compressing systems (Figure 6). For these systems, a known pseudo-noise signal is multiplied by the carrier signal. This method is chosen always to achieve better ranging accuracy because of the limited bandwidth in real applications. Lastly, UWB, ultra-wide band, is the latest and a more accurate and promising method [4]. In this method,

(5) $$d=\frac{c}{BW}$$

where *d* is the absolute resolution and BW is the bandwidth of the signal. The large bandwidth of the UWB system is capable of resolving multiple paths and combating multipath fading and interference. However, these systems are limited to low range and building penetration by large attenuation. One of the main problems of UWB systems is the interference between UWB devices and other services, such as GPS systems, operating at 1.5 GHz.

In addition, the authors in [26] use a mobile sensor unit for ToF-based measurements and take into account the effect of the electrical properties of different organs and tissues. For this purpose, they divide the human body into four sub-volumes and calculate the average relative permittivity value for each region. However, this method does not provide precise data on the relative permittivity of the human body.

The study in [4] compares the effect of the number of capsules and sensors on the localization accuracy and demonstrates that for both ToF and RSS approaches, the number of receivers on the body surface has more effect on the accuracy of positioning than the number of capsules in cooperation in the GI tract, based on both ToF and RSS methods.

### 2.3. AoA-Based Techniques

The accuracy of an AoA measurement system is determined by the resolution of the directional antenna or antenna array and the algorithms used for estimating the AoA simultaneously [7]. With the exception of AoA-based methods, the first step in the localization process is the estimation of the distance (also called ranging) from the transmitter to the different receivers. Hence, a more accurate distance estimate will result in a more accurate position estimate.

**Figure 7:**
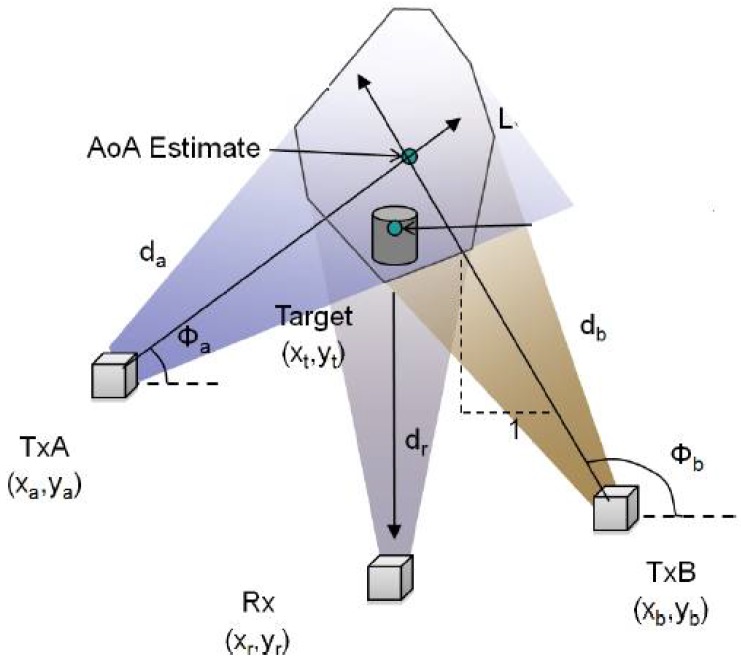
The idea behind the AoA-based technique: the AoA is calculated representing the direction in which the signal is emitted.

Given the accuracy of AoA measurement system, the number of reference points is determined by the target position with respect to the reference points when the target lies between the two reference points (Figure 7). AoA measurements will not be able to provide the exact location of the target on the line between the two reference points. Hence, more than two reference points are required to have more accurate data on the localization [7].

### 2.4. RFID-Based Techniques

Besides the RSS technique, RFID is also investigated in RF-based localization systems for WCE [27,28,29,30,31]. Here, a cubic antenna array is placed around the human body to detect an RFID tag placed inside a capsule. Similarly, in [28], a cubic antenna array is designed around the body to sense the RFID tag inside the WCE. Localization is performed based on the assumption that the closest antenna detects the tag. An improved method consisting of RFID tags having bi-directional antennas is presented in [29,31]. The phase difference of the signal from an RFID tag without any localization algorithm is discussed in [27]. Using this approach, the authors in [30] use support vector regression to sense bio-medical sensors/devices, such as needles and catheters, having an RFID tag with a mean accuracy in the millimeter range. However, the tag orientations are kept fixed, and the effect of the human tissues on the RF signal is not taken into account.

## 3. Magnetic-Signal-Based Localization and Tracking

In magnetic signal-based methods, a permanent magnet is united with the biomedical sensor, and an exterior array of magnetic sensors is located outside the human body. Since the biomedical sensor moves together with its magnet, magnetic-signal-based methods have the advantages of magnetic levitation, robotic magnetic steering, helical propulsion by a rotational magnetic field and remote magnetic manipulation [67,68,69]. Hence, a considerable amount of research is focused on building active locomotion biomedical sensors and their settings [34,44,70]. The main advantage of positioning techniques based on the magnetic field strength is that low-frequency magnetic fields can run through the body with reduced attenuation since tissues of the human body are non-magnetic. However, one challenge is the interference from the magnetic fields produced by the materials present in the environment and also from the Earth’s magnetic field, and this may require additional hardware for the analysis of the magnetic signal to solve the positioning problem [7].

A series of studies [37,67,70] have proposed a pioneer magnetic strength-based localization algorithm for WCE. In these studies, the authors used a WEC with an interior magnet and placed tri-axial magnetic field sensors outside the body to estimate the WEC’s location. Alternatively, in [12], the implementation required additional hardware. This encouraging technique to track the movement of a WEC through the GI tract is through the magnetic strength of an on-board permanent magnet [7]. Magnetoresistive sensors bonded to the skin allow the positional error of the 6D location data of the WEC to be around 3.3 mm [71]. However, magnetic signal-based techniques have some drawbacks, as well, including inconvenient weight and size, conflicts between actuation and localization systems, certain health risks for patients associated with increased magnetic field and magnetic field interference with other magnetic field sources, such as MRI systems [7].

There exist recent works on the compatibility of WCE with implanted biomedical devices, such as cardiac pacemakers, and non-medical devices, such as mobile phones and laptops [52]. In the experimental tests of [52], devices were activated alone or simultaneously in proximity with another device. The functioning of the WCE may also be affected by metal clips, batteries, magnets in the body and the nearby surroundings. Further research is required to quantify the effects [52].

Additionally, some magnetic capsules (Navi Capsule, IntroMedic) have been approved by South Korea to help with mobilizing the device through the GI tract to facilitate delivery in patients with delayed gastric emptying [3]. Furthermore, the safety of the mechanical aspects of localization, the magnetic fields produced by the neodymium iron boron (NIB) magnets used in the study [72], with a magnetic field of up to two Tesla, are classified as non-significant risk devices by the USA Food and Drug Administration (FDA).

In the implementation and application of the magnetic field-based techniques, magnetic signals are used for both detecting the location of diseases and as a feedback mechanism for the actuation system. Further, significant interference exists between the localization and actuation systems [37,70,71,73]. Hence, these two systems have to be considered together. In the literature, based on the actuation system, magnetic-field localization is studied separately for passive and active magnetic manipulation of biomedical sensors.

### 3.1. Magnetic Localization and Tracking of Passive Sensors

**Figure 8:**
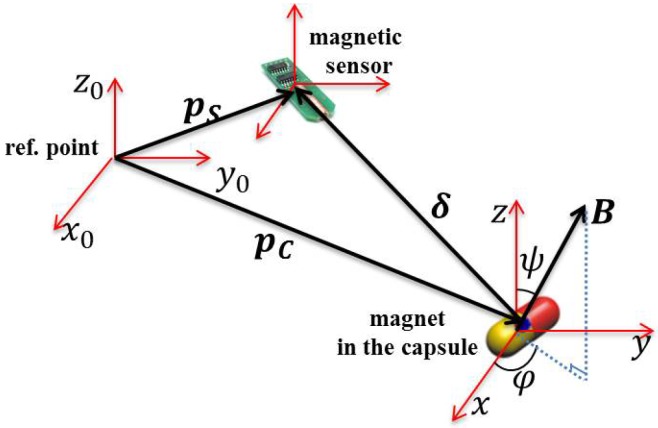
Coordinate frame of a magnet for WBC localization.

In a magnetic-field-based localization scheme, the magnetic source and the magnetic sensor modules are the most significant elements (Figure 8). The magnetic source inside the biomedical sensor can be formed in three different ways, using: (i) a permanent magnet; (ii) an embedded secondary coil; and (iii) a tri-axial magnetoresistive sensor [7,37].

Most researchers focus on the use of a permanent magnet inside the WBS in the literature, since this approach provides the generation of a magnetic field, and based on the magnet position and orientation, magnetic sensors placed outside the patient’s body can detect the magnetic flux intensities [6,7,34,39]. The magnet position and orientation can be computed by feeding the sensor data to an appropriate algorithm [37,38] based on the well-established mathematical model of the magnetic field of a magnet with position ${\left[a,b,c\right]}^{T}$ and orientation $H_{0}={\left[m,n,p\right]}^{T}$, at a certain point ${\left[x,y,z\right]}^{T}$, given by:

(6) $$B=\frac{\mu_{0}\mu_{T}M_{T}}{4\pi }\left(\frac{3(H_{0}^{T}P)P}{{∥P∥}^{5}}-\frac{H_{0}}{{∥P∥}^{3}}\right),$$

(7) $$M_{T}=\pi \sigma^{2}LM_{0},$$

where $P={\left[x-a,\;y-b,\;z-c\right]}^{T}$ is the relative position of the magnet. The $\mu_{T}$, $\mu_{0}$, $M_{T}$, *σ*, *L* and $M_{0}$ are the relative permeability of the medium, the air magnetic permeability, magnetic intensity of the magnet, the radius of the magnet, the length of the magnet and the magnetization strength, respectively.

In [40], a three-magnet positioning method is introduced to eliminate the interference caused by the complicated structure of the human body during the localization process using a magnetic flux density-based algorithm and a sensor array with tri-axial magnetic sensors. The study in [44] proposed an original approach where the magnetic field sensors are placed in a biomedical sensor for positioning by considering a pre-computed magnetic field model together with the sensed data.

The authors of [45] introduce a 3D localization method for the magnetically-actuated soft WEC using a coaxially-aligned exterior permanent magnet. Estimated distance, depending on the WEC shape deformation as the exterior magnet gets closer to the body, helps to track the WEC inside the body.

The study in [41] introduces a technique to measure the magnetic field generated by an exterior magnet at the center of an interior magnet placed in a WEC, by eliminating the interaction of the interior magnet. Data on such magnetic fields enable the manipulation of the magnetic field around the body and help to control the motion of the WEC. The article [36] proposes a non-iterative positioning technique by applying a rotating magnetic dipole to create highly accurate 6-DoF position data. However, the technique requires a 30 s post-processing time.

Another magnetic field-based WEC localization method is proposed in [42], utilizing sensed data and pre-defined magnetic field model. This method provides 6-DoF location and orientation data and can be implemented in real-time during the actuation of the WEC using an exterior permanent magnet. The authors further improve this algorithm in their later studies by using the Jacobian of the magnetic field of the capsule to eliminate magnetic dipole assumption inaccuracies [43].

### 3.2. Magnetic Localization and Tracking of Active Sensors

These systems are developed to function efficiently with their own magnetic actuation. Accordingly, many research groups are investigating the design and development of active locomotion sensors and settings [7,32,33,34].

#### 3.2.1. Alternating Magnetic Field-Based Techniques

This technique uses a spiral structure-shaped permanent magnet on a capsule that is incorporated with three pairs of coils, located in three perpendicular axial directions to create an exterior rotating magnetic field around the human body. The spiral pattern rotates the capsule using the magnetic field around the capsule and can move the capsule back and forth. The frequency of the rotational magnetic field cannot exceed the 10 Hz limit [7].

#### 3.2.2. Inertial Sensing-Based Techniques

The inertial “magnetic steering” technique utilizes a 6-DoF robotic arm that has a permanent magnet at its end effector. Four cylindrical magnets are placed over the surface of the biomedical sensor to form a magnetic link between the sensor surface and the exterior permanent magnet so that the capsule can be drifted and directed efficiently through the magnetic interaction. For localization purposes, a tri-axial accelerometer is inserted into the capsule [7].

#### 3.2.3. Exterior Rotational Magnetic Field-Based Techniques

These techniques utilize a helical architecture for the WEC, which generates an exterior rotational magnetic field to rotate the two permanent magnets placed in the WEC [35,36]. In [35], a large parallel piped permanent magnet composed of seven smaller rectangular magnets is rotated to create a magnetic field. Here, an electrical motor mounted on a manipulator helps to generate the magnetic field, since it can rotate and its location can be altered during the motion control of the WEC [7].

## 4. Localization and Tracking Algorithms

The previous two sections have focused on the RF electromagnetic and magnetic signal-based distance and/or bearing measurement techniques utilized in biomedical sensor localization and tracking. Such distance and/or bearing measurements need to be effectively fused using suitable algorithms to produce an on-line (real time) location estimate of the biomedical sensor. The distance and/or bearing measurement-based localization algorithms in the literature can be categorized as linear vs. nonlinear algorithms and off-line/batch vs. on-line/adaptive/recursive algorithms [39,61,63,65,74].

The study in [39] indicates that nonlinear algorithms for magnetic sensing-based methods have some disadvantages, such as their slow speed, computational complexity and dependence on the initial parameter estimates. Linear algorithms can provide better solutions in terms of rapidity and achieving a real-time tracking system.

There exist several minimization algorithms studied for localization applications, such as Powell’s algorithm [75], the downhill simplex algorithm [75], DIRECT [39], multilevel coordinate search (MCS) [76] and the Levenberg–Marquardt method [77], to solve the high-order nonlinear localization equations. The Levenberg–Marquardt method is used in [19,37,38,39,67,70] for WEC localization and orientation. The trilateration method is another localization approach to estimate the WBS position, based on the transmitter-receiver distances in a sensor network. Proximity data from the measurement units are converted into position information generally by applying triangulation that takes the features of distance geometry and rigid graph theory into account [63]. Further varieties and details of wireless sensor network-based localization algorithms utilizing distance and/or bearing measurements can be found in [63,78,79].

There are various positioning algorithms and techniques proposed in the literature to find the location and orientation of a sensor in the human body. Among these methodologies, RF signal-based location estimation methods are favourable based on their implementation and cost effectiveness. Accordingly, such RF-based techniques have been utilized in the SmartPill, MicroCam and the M2A biomedical capsules [4]. ToF-based methods are well known to provide higher accuracy in comparison to RSS- and AoA-based methods. However, the intense absorption of the human body leads to large errors in the ToF estimate. Furthermore, the limited bandwidth (402–405 MHz) according to the Medical Implant Communication Services (MICS) prevents very accurate ToF measurements. In addition, because of relative permittivity uncertainties and variations in the human body, large errors are observed. The peristalsis action of the human body causes even more unpredictable distance measurement errors [4].

Another WBS localization and tracking approach studied in the literature is adaptive localization and tracking [6,22,47,61] based on linear parametric modelling [80] of the governing sensor equations and some other adaptive control tools [74,80]. The approach in [6,22,47] utilizes geometric cooperative sensor methods [61] to estimate the path loss coefficient for RSS and the relative permittivity for ToF-based methods, using a mobile sensor triplet instead of a single sensor. It involves an adaptive localization and tracking scheme integrating this coefficient estimation technique and a discrete-time recursive least squares (RLS) parameter identification algorithm.

## 5. Hybrid Localization and Tracking Techniques

Existing commercially available RF-based positioning systems can only provide low accuracy and discrete position estimates of the WCE location and/or orientation, due to the complicated structure of the body tissues and highly complicated geometry of the GI tract. As an alternative approach, hybrid techniques, including fusion of RF electromagnetic signal and video-based techniques [46], fusion of RF electromagnetic signal and magnetic-field-based techniques [6] or fusion of other sensing modalities, have the potential to provide more accurate simultaneous location and orientation estimates. Although these hybrid techniques naturally require the integration of multiple technologies and have higher algorithmic complexity, using hybrid methods accordingly has some capabilities to provide higher accuracy on the localization problem of implantable or wearable medical devices within the safety limits, since each of the aforementioned methods has some limitations due to the safety regulations, such as MICS (402–405 MHz).

A particular approach to further improve the WEC positioning accuracy and generate a 3D map of the GI tract is to obtain the estimates using different types of techniques independently first and then effectively fuse the generated estimates of the WEC position [13].

### 5.1. RF and Video Fusion-Based Techniques

The authors of [13,46] propose a hybrid WEC localization system that integrates the RSS-based (or ToF) RF positioning with the image processing-based tracking of the WEC. In these articles, the problem is mathematically analysed and the corresponding accuracy level is derived in terms of the Cramer–Rao lower bound for the proposed hybrid WEC localization system. The design and analysis are established for both RSS and ToF distance measurements.

### 5.2. RF and Magnetic Strength Fusion-Based Techniques

In [6,47], the authors have investigated WBS tracking, for magnetic sensing and actuation settings, where an embedded permanent magnet is used inside a passive WBS together with magnetic sensors outside the body, producing a magnetic field around the WBS. In those studies, a hybrid localization technique with high accuracy for simultaneous location and orientation estimation has been introduced. The proposed hybrid localization technique is based on data fusion of magnetic measurements and electromagnetic signals emitted by the WBS for image transmission and other medical information using a similar adaptive RLS parameter estimation scheme. This method provides higher accuracy with relatively low mathematical complexity and a smaller number of magnetic sensors, since the applied adaptive tracking law enables the distance between the magnetic sensor and the capsule in a certain sensing range [13].

### 5.3. Magnetic Strength and Image Fusion-Based Techniques

The authors in [48] introduce an ultrasound imaging-based localization scheme for the WECs, integrated with magnetic-field-based localization. The localization system is composed of a Cartesian robot actuating a transcutaneous sonographic probe in 2D. While the WEC moves along the GI tract via the use of a magnetic field, the localization system generates sonographic image data through the back of the person, illustrating the current position of the WEC. The results provided in [48] verify that the approach is real-time implementable and can be applied to navigate a WEC inside the patient’s body.

## 6. Other Techniques

As alternatives to the aforementioned localization and tracking methods, computed tomography (CT), X-rays, MRI or *γ*-rays can be used for localizing a WBS inside the GI tract by inserting radiation opaque material into the WBS [7,51,53,54]. However, using these techniques is costly, and there exist some health risks for the patient [7]. For instance, the study in [52] indicates that undergoing magnetic resonance imaging (MRI) while the WCE is inside the patient’s body might cause serious damage to the patient’s GI tract.

Ultrasound sensing is another alternative technique for localization in soft tissues [81]. In this approach, the implantable biomedical sensor position information is estimated using the ToF measurements between the ultrasonic signals transmitted from an exterior source and the signals reflected by the capsule [81]. Here, accurate data on the speed of sound in human tissues is essential for accurate tracking. Furthermore, the WBS is required to stay in the scanning plane to be sensed [50]. These two constraints can be relaxed following a second approach, where an ultrasound transducer embedded in the biomedical sensor emits ultrasonic signals to be received by exterior receivers placed over the patient’s abdomen [7,50].

Another positioning technique based on microwave imaging is introduced by [49]. In [49], electric features of different tissues and organs are considered together with different tissue and organ locations to help to acquire more accurate data on the 2D position of the WBS. Preliminary tests resulted in errors less than 1 cm in 2D.

## 7. A Discussion on the Connection and Cooperation with Wearable Sensors

In this section, we discuss the connection and cooperation between implantable biomedical sensors (IBSs) and wearable/body sensors from two perspectives: (1) the connection between IBS and wearable sensor localization and tracking schemes; and (2) the cooperative use of IBSs and wearable sensors in hybrid sensor network settings, where the IBS localization and tracking is performed in cooperation with the wearable sensors in the network.

From the first perspective, note that the second major class of biomedical sensors, complementary to implantable ones is the class of wearable sensor systems. With their low software and hardware costs and requirements, light weight, compactness and portability, wearable units have become a significant alternative to cameras or other external sensor systems embedded to the environment. In particular, wearable biomedical sensor technologies provide lower cost of care, minimally-invasive and effective procedures and shorter recovery times that improve the health outcomes. Detailed characteristics and various applications of wearable biomedical sensor systems can be found in [57,82,83,84,85,86]. Some of the more recent wearable sensor application areas are monitoring of vital signals, medical diagnosis and treatment, home-based rehabilitation and physical therapy, telesurgery, biomechanics, gait and posture recognition, detecting the emotional state and stress level of people and remote monitoring of the physically or mentally disabled, the elderly and children.

Wearable motion sensors, such as accelerometer-, gyroscope- and magnetometer-based ones, are widely used in monitoring the activities of daily living [87,88,89,90] and the detection of anomalies, such as falls. Falls are potentially dangerous and often occur unexpectedly while performing daily activities or when making transitions between two activities that require a change of body posture. Falls might lead to serious injury or even death if medical attention cannot be provided rapidly [91,92,93]. For these reasons, they should be detected, classified and localized reliably to reduce fall-related injuries. Smart phones that contain embedded accelerometers and gyroscopes are suitable devices for executing fall detection algorithms [94]. In a typical scenario, a small network for the user is pre-defined on his/her telephone. This network may consist of a hospital, a call center, healthcare professionals, the user’s relatives and friends, etc. If the algorithm detects a fall, a warning or alarm signal can be automatically sent to this small network to alert them about the fall. This important feature can be complemented by biomedical sensors that monitor vital symptoms, such as the heart rate, blood pressure and body temperature.

It is also crucial to accurately identify the location where the fall took place, so that medical help can be provided rapidly. Remote health monitoring and fall detection systems combine these tasks with the localization of the person, who has vital symptoms or has fallen, via one of the aforementioned positioning methods [84]. Mobile devices that run fall detection algorithms provide a suitable medium for this purpose, as well. Localization and activity recognition can be performed simultaneously within the same loop, in which localization accuracy can be improved using activity cues [95] and vice versa. Since these tasks also require wireless technology to acquire and transmit data, approaches similar to those in the previous sections can be employed for localization.

Most of the techniques reviewed in the previous sections on the localization and tracking of IBS systems can be adapted to wearable sensor systems, as well. However, the use of these technologies is currently limited to highly controlled laboratory environments and clinical trials. The studies in [96,97,98] indicate that the positioning of wearable motion sensors with embedded wireless connectivity is important to achieve more accurate results. However, in the literature, optimization of the positioning of wearable sensors is not well studied [99], and there exist some contradictory results. Another difficulty is the variability of the acquired data between the different subjects and experiments [100].

From the cooperation perspective, wearable sensors can be used in conjunction with IBS networks to allow people to be monitored continuously during their everyday activities. Modern IBS systems apply wireless technology for data acquisition and transmission. Wearable sensor systems can also cooperate with IBS systems synchronously [6,22,101]. To this end, wearable sensor systems need to be placed at well-defined positions on the human body to transmit the collected data outside the body to health professionals, caregivers, family members or a monitoring device, such as a cell phone or computer. However, one of the main issues is deciding how and where to position these sensor nodes and how many of them to use, in a form wearable on the human body, to enable more efficient and accurate measurement and transmission [7,22,96,98,101]. In a recent study [102], the authors propose two algorithms that are invariant to how wearable motion sensors are oriented on the body at fixed positions in the context of activity recognition. This should soon be followed by position-invariant algorithms to allow some flexibility in the placement of wearable sensors.

Further, in the implementation of some of the IBS localization and tracking schemes covered in the previous sections, especially the sensor network-based cooperative ones, wearable sensors are utilized. For example, the RSS-based IBS localization setting described in Section 2.1 and illustrated in Figure 4 utilizes wearable sensors that are composed of electromagnetic signal receivers, processors and transmitters for the transmission of the collected information to central monitoring units. Similarly, in the magnetic signal-based IBS localization and tracking schemes described in Section 3, an exterior array of magnetic sensors is utilized as a wearable sensor network.

## 8. Conclusions

In this article, the existing literature on the localization and tracking of robotic implantable sensor systems has been reviewed. The main localization and tracking methodology approaches, with their merits and limitations, have been presented. For these approaches, both the current state-of-the-art commercial products and system designs within the research domain are summarized. In all of these approaches, there exists a significant amount of open research tasks for further investigation in terms of improving positioning accuracy and practical implementation. Further, a short review of extensions and counterparts of such techniques for wearable biomedical sensor systems has been provided. In current implantable biomedical sensor design studies, one goal is to have full robotic capabilities for simultaneous diagnosis and treatment. In order to achieve this goal, developing hybrid techniques is a promising approach for accurate localization within the safety limits. Motion control of biomedical implantable sensors is another key research topic for developing fully robotic implantable sensor systems for drug delivery, surgery and many other biomedical applications.

## Figures and Tables

**Table 1:** Implantable biomedical sensor localization techniques in the literature.

Implantable Biomedical Sensor Localization Techniques										
RF Electromagnetic Wave Based Techniques				Magnetic Field Strength Based Techniques		Hybrid Techniques			Other Techniques	
RSS [4,12,18,19,20,21,22,23,24,25]	ToF and TDoA [4,18,26]	AoA [7]	RFID [27,28,29,30,31]	Active [6,32,33,34,35,36]	Passive [6,34,36,37,38,39,40,41,42,43,44,45]	RF and Video [13,46]	RF and Magnetic [6,13,47]	Magnetic and Video [48]	Ultrasound, MRI, CT [49,50,51,52,53,54]	X-Ray, *γ*-Ray, Visible Wave [51,53,54]
